# Polarization-encoded quantum key distribution with a room-temperature telecom single-photon emitter

**DOI:** 10.1093/nsr/nwaf147

**Published:** 2025-04-16

**Authors:** Xingjian Zhang, Haoran Zhang, Rui Ming Chua, John Eng, Max Meunier, James A Grieve, Wei-Bo Gao, Alexander Ling

**Affiliations:** Centre for Quantum Technologies, National University of Singapore, Singapore 117543, Singapore; Division of Physics and Applied Physics, School of Physical and Mathematical Sciences, Nanyang Technological University, Singapore 637371, Singapore; Centre for Quantum Technologies, National University of Singapore, Singapore 117543, Singapore; Quantum Research Center, Technology Innovation Institute, Abu Dhabi, United Arab Emirates; Division of Physics and Applied Physics, School of Physical and Mathematical Sciences, Nanyang Technological University, Singapore 637371, Singapore; Division of Physics and Applied Physics, School of Physical and Mathematical Sciences, Nanyang Technological University, Singapore 637371, Singapore; Quantum Research Center, Technology Innovation Institute, Abu Dhabi, United Arab Emirates; Centre for Quantum Technologies, National University of Singapore, Singapore 117543, Singapore; Division of Physics and Applied Physics, School of Physical and Mathematical Sciences, Nanyang Technological University, Singapore 637371, Singapore; School of Electrical and Electronic Engineering, Nanyang Technological University, Singapore 637371, Singapore; Centre for Quantum Technologies, National University of Singapore, Singapore 117543, Singapore; Department of Physics, National University of Singapore, Singapore 119077, Singapore

**Keywords:** single-photon source, quantum key distribution, polarization, polarization mode dispersion

## Abstract

Single-photon sources (SPSs) are directly applicable in quantum key distribution (QKD) because they allow the implementation of the canonical BB84 protocol. To date, QKD implementations using SPSs are not widespread because of the need for cryogenic operation, or frequency conversion to a wavelength efficiently transmitted over telecommunication fibers. We report an observation of polarization-encoded QKD using a room-temperature telecom SPS based on a GaN defect. A field test over 3.5 km of deployed fiber with 4.0-dB loss yielded a quantum bit error rate (QBER) of 5.0% and a secure key rate of 585.9 bps. Further testing in a 32.5-km fiber spool (attenuation of 11.2 dB), which exhibited substantially lower polarization mode dispersion, yielded a QBER of 3.2% and a secure key rate of 50.4 bps. These results illustrate the potential of the GaN defects for supporting polarization-encoded quantum communication.

## Teaser

This work explores quantum key distribution over deployed fiber using a room-temperature telecom single-photon source, paving the way towards realistic implementations of single-photon-based quantum communication.

## INTRODUCTION

The original quantum key distribution (QKD) protocol by Bennett and Brassard (BB84) proposed that two parties sharing single photons could generate a secure encryption key [1]. Many QKD works use weak coherent pulses [2–4] along with decoy state techniques [5–8] because they are easy to implement. Although impressively high key rates and long distances [9] have been achieved with these sources over fiber and free space, performing QKD with single-photon sources (SPSs) remains appealing due to reduced engineering overhead, and a reduction in potential security loopholes [10–14].

Many materials have been investigated as solid-state SPSs. Examples include semiconductor quantum dots [15–20] and nitrogen-vacancy color centers [21,22]. Reliable telecom-band SPSs are particularly desirable for QKD applications due to their compatibility with existing deployed fiber links. However, most SPSs emit at wavelengths shorter than the telecom band [23–25], where dedicated fibers [26] or additional frequency conversion steps [27,28] are required. Moreover, many telecom-band SPSs require cryogenic cooling systems [29–32], limiting their commercial feasibility for QKD. Alternatively, SPSs outside the telecom band, such as hexagonal boron nitride, which also operate at room temperature, could enable QKD over free-space links [33,34], although they are not compatible with fiber-based communication. On the other hand, a room-temperature SPS within the telecom band [35] would be an excellent quantum source for deployed QKD [36].

Here we report an implementation of polarization-encoded QKD using a room-temperature, telecom-band-compatible SPS based on a GaN defect. The SPS produces single photons within the telecom O-band, centered at 1309.5 nm, and was used to demonstrate the BB84 protocol. A field trial was first performed over a 3.5-km deployed fiber loop with a loss of 4.0 dB. The effects of polarization mode dispersion were minimized by carefully selecting the appropriate polarization states for transmission. The quantum bit error rate (QBER) was observed to be 5.0%, with a secure key rate of 585.9 bps achieved through a specially optimized, unbalanced basis selection probability. Additional testing on a 32.5-km fiber spool yielded a secure key rate of 50.4 bps, suggesting the potential for this SPS in polarization QKD over longer distance. Our experiment demonstrates the feasibility of GaN-based room-temperature telecom SPSs for polarization QKD in deployed fiber links.

## RESULTS

### Single-photon generation

**Figure 1.**
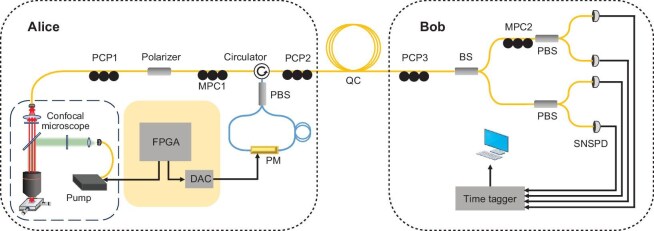
Experimental setup for SPS BB84. PCP, polarization controller paddles; MPC, motorized polarization controller; PM, phase modulator; QC, quantum channel; BS, beam splitter; PBS, polarization beam splitter; SNSPD, superconducting nanowire single-photon detector; FPGA, field-programmable gate array; DAC, digital-analog converter. The yellow line represents the single-mode fiber, while the blue line represents the polarization-maintaining fiber.

In this experiment, the sample is a GaN thin film grown on the patterned sapphire substrate (PSS) [35]. The emitters are randomly distributed on the sample. An oil immersion confocal microscope was used to both optically pump (1064 nm) as well as collect the luminescence above 1200 nm using appropriate filtering. An overview of the setup is depicted in Fig. 1. All the experiments in this work were completed with the sample under ambient laboratory temperature, without any dedicated temperature control.

**Figure 2.**
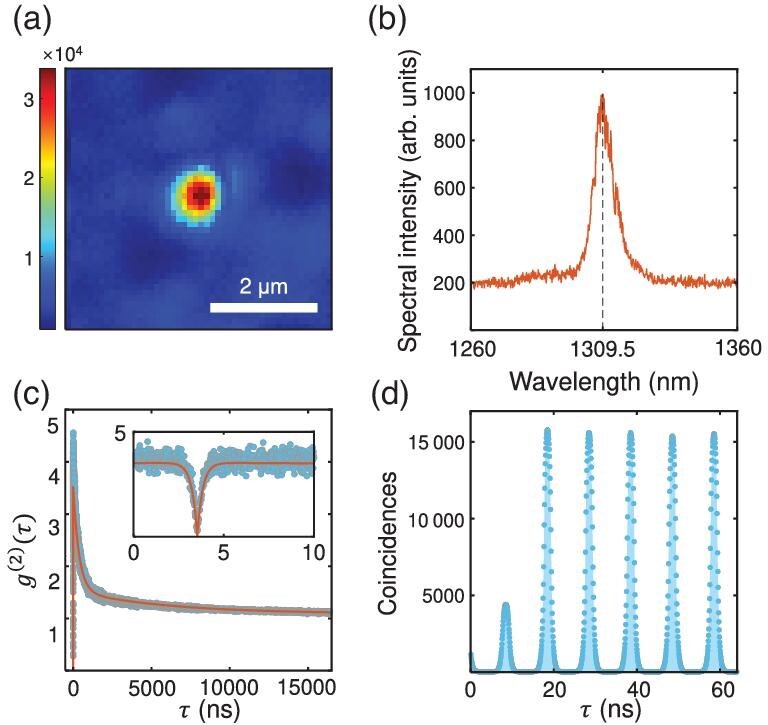
The optical characterization of the GaN SPS emitter sample used in this experiment, taken with a 1200-nm long-pass filter and a 1000-nm dichroic mirror. (a) The spatially resolved confocal map of the emitter. The structure of the patterned sapphire substrate is visible in the background. (b) The photoluminescence spectrum of the sample emitter when the pump wavelength is 1064 nm. (c) The $g^{(2)}(\tau )$ data obtained using 0.1-mW continuous pump (blue dots), fitted with the three-level model (orange line). (d) The measured $g^{(2)}(\tau )$ obtained using a 100-MHz pulsed pump with a width of 80 ps.

A bright defect-based emitter is located within the GaN layer, with an emission wavelength centered at 1309.5 nm to reduce the loss and dispersion in the telecom fiber. The PSS increases the extraction efficiency of the emitted photons, and therefore the detected count rate [35]. A confocal map around the emitter and a photoluminescence spectrum are shown in Fig. 2a and b, respectively.

The SPS quality was qualified by measuring the second-order correlation function $g^{(2)}(\tau )$ under continuous and pulsed conditions. Figure 2c shows a measured $g^{(2)}(0)$ of $0.28 \pm 0.04$ with continuous pump. With a pulsed pump (80-ps duration), the pulsed $g^{(2)}(0)$ was measured to be $0.323\ \pm \, 0.005$, as shown in Fig. 2d. This $g^{(2)}(0)$ value indicates that the emitter exhibited low multi-photon possibility and that most of the signal will not be lost during the key distillation phase.

### Polarization mode dispersion in quantum channels

A four-state polarization BB84 protocol was implemented in this experiment. Because of the relatively broad bandwidth (7 nm) of our quantum source, polarization mode dispersion (PMD) has been identified as a major factor affecting the QBER. In addition to causing a small difference in the arrival time between different polarization modes, PMD in the quantum channel can have a significant effect on the QBER of polarization-encoded protocols—a consequence of changes in the output polarization against wavelength [37–39]. For any small bandwidth where the first-order PMD is dominant, the effect could be visualized as the outgoing state tracing along an arc centered around a rotation axis on the Poincaré sphere. A pair of polarization states, known as the principal states of polarization (PSPs), can thus be found along the rotation axis. These states remain unaffected by first-order PMD and can be used to minimize its impact in this experiment. The total PMD vector can be expressed in the form of a Taylor series:


(1) \begin{eqnarray*}
\boldsymbol {\tau }(\omega )=\boldsymbol {\tau }(\omega _0)+\frac{d\boldsymbol {\tau }}{d\omega }\Delta \omega +\cdots .
\end{eqnarray*}

Here $\boldsymbol {\tau }(\omega _0)$ represents the first-order PMD vector. Its magnitude corresponds to the delay between the two PSPs, or, in other words, the value of the differential group delay, with the direction pointing toward the slower PSP [40]. The higher-order terms in the series describe how the PMD vector varies with wavelength.

**Figure 3.**
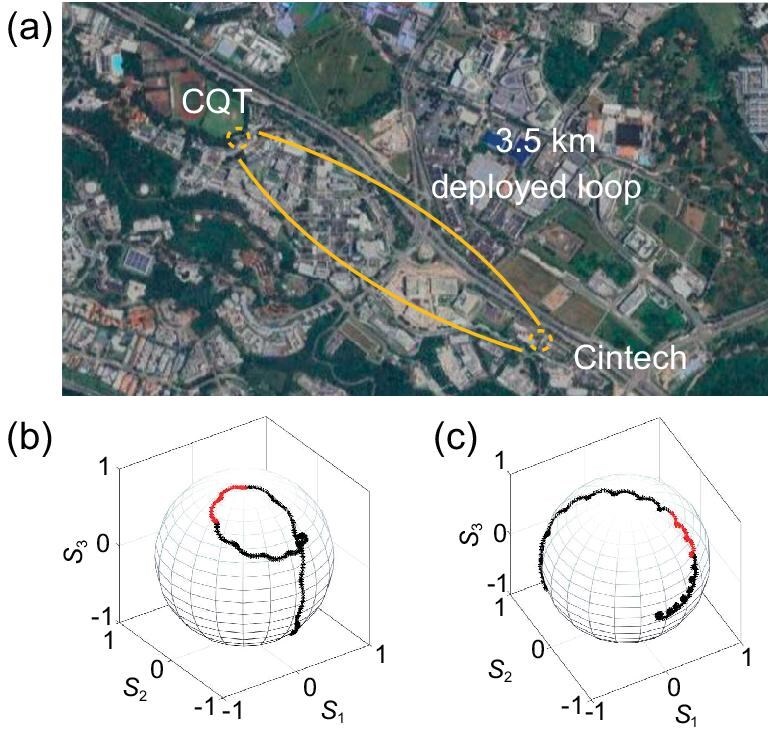
Illustration of the deployed channel and the PMD characterization. (a) Map of the start and end points of the deployed channel. (b, c) The spread in polarization due to PMD in the (b) deployed channel and (c) spool. The wavelength range was 1280–1340 nm. The wavelength band of the photons from the SPS ($1309.5 \pm 3.5$ nm) is marked in red.

The effect of the fiber channel–induced PMD could be visualized by sending a narrow-band tunable laser and tracing the polarization trajectory on the Poincaré sphere against the wavelength. We conducted tests on a 3.5-km deployed fiber as in Fig. 3a and a 32.5-km fiber spool. The results plotted in Fig. 3b and c depict how a fixed polarization state distributed over long fiber is dispersed in a broad wavelength range (60 nm). The polarization-wavelength trace in deployed fibers typically exhibits more irregular shapes than in spools due to the increased influence of higher-order PMD. The trajectory is also revealed to be reasonably close to an arc within a small wavelength range, where the higher-order terms have a minor effect.

**Figure 4.**
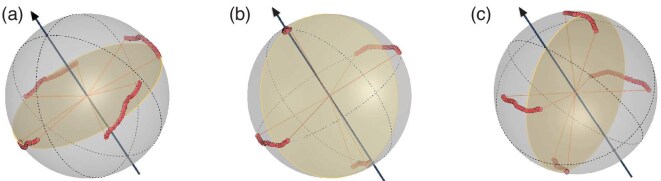
The polarization trajectories (red dots), when starting at different locations on the Poincaré sphere relative to the PMD vector (black arrow). This is for a wavelength range of 7 nm and measured after the photons travel over 3.5 km of deployed fiber. Here we show three representative choices for the four states used in the BB84 QKD protocol that can influence the final QBER value. (a) The special case when the four states are all orthogonal to the PMD vector. All states suffer a maximum scattering. (b) The case when two of the states are orthogonal to the PMD vector. Only two states experience maximal scattering. (c) All the states have equal but reduced scattering.

It has been shown that selecting the appropriate basis sets can reduce the impact of PMD [41] by half compared to the worst case. The impact of first-order PMD on the Poincaré sphere is described by


(2) \begin{eqnarray*}
\Delta \theta =|\boldsymbol \tau |\Delta \omega ,
\end{eqnarray*}

where $\Delta \theta$ is the field angle of the scattering trace. Therefore, a minimum total scattering could be achieved if the PMD vector sits on the same great circle as the four states of polarization QKD. We demonstrated this experimentally in Fig. 4. According to the results, states closer to the PSP experience less impact from PMD. A poor choice, as shown in Fig. 4a, results in $\Delta \theta$ scatter for all four states, while better choices can reduce the QBER by setting no scatter for two of the states in (b) or ${\Delta \theta }/{\sqrt{2}}$ for all of them in (c). Table 1 lists the central angles corresponding to each arc trajectory in Fig. 4. Here $|0\rangle$, $|1\rangle$, $|+\rangle$ and $|-\rangle$ represent the polarization states commonly used in BB84 without loss of generality. To achieve the minimum influence of PMD, the great circle defined by these four states should intersect the PSP vectors. Case (b) is applied in our experiment.

**Table 1.** Central angles of each state.

State	Case (a)	Case (b)	Case (c)
$|0\rangle$	51.5$^\circ$	12.9$^\circ$	39.6$^\circ$
$|1\rangle$	51.6$^\circ$	14.1$^\circ$	40.8$^\circ$
$|+\rangle$	50.4$^\circ$	66.5$^\circ$	24.2$^\circ$
$|-\rangle$	52.8$^\circ$	55.1$^\circ$	59.0$^\circ$
Average	51.6$^\circ$	37.2$^\circ$	40.9$^\circ$

The magnitude of $\Delta \theta$ could be determined by observing the scatter trajectory of two sets of orthogonal polarization bases against the wavelength, as illustrated in Fig. 4. The PMD value of the link could thus be estimated by calculating $\Delta \theta /\Delta \omega$ [42,43]. The deployed link exhibited a higher PMD parameter of $0.072\, \text{ps}/\sqrt{\text{km}}$, when compared with $0.021\, \text{ps}/\sqrt{\text{km}}$ observed in the fiber spool, which became a major source of our QBER. The difference may be attributed to aging, advancements in manufacturing technology that have reduced PMD, as well as the lower PMD observed in the uncabled fiber compared to the cabled fiber [44].

### Key rates

**Figure 5.**
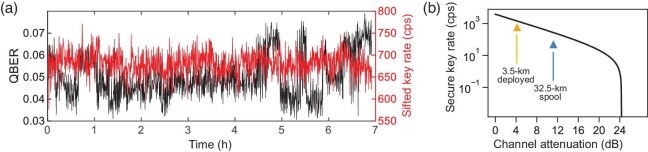
Results of the QKD experiment over quantum channels. (a) The record of the average QBER and sifted key rate over the deployed channel within 7 h. (b) The experimental results over different quantum channels compared with the GLLP bound based on our current devices.

Figure 5a records the variation in the QBER and sifted key rate for signals transmitted over the deployed fiber, with an integration time of 20 s. The D and A polarizations were pre-aligned to the PSPs of the channel, as previously mentioned. This yielded a QBER of 1.7% for the D/A basis and 8.3% for the L/R basis, averaging 5.0%. An averaged raw key rate of 1349.6 bps was achieved over a 7-h duration without active feedback. With the experimental parameters of the devices and the assumed variables summarized in Table 2, we carried out a finite-key analysis to bound the amount of secure key to be $\epsilon _{\rm sec}$ secret and $\epsilon _{\rm cor}$ correct [45]. In this experiment, the DA and LR bases had an equal preparation and measurement probability, which resulted in a secure key rate of 247.3 bps under the finite-key analysis. However, considering the nature of the unbalanced QBER caused by PMD, an unbalanced ratio of bases could be designed to maximize the key rate. The secure key rate could be increased to 585.9 bps by using a preparation probability of $p_z=0.997$ and $p_x=0.003$. This well approaches the asymptotic limitation from GLLP [46,47] for the experimental parameters used in this work, as shown in Fig. 5b. The result for the 32.5-km spool is also indicated with a reasonably low QBER of 3.2% and a secure key rate of 50.4 bps.

**Table 2.** Overview of the parameters.

	Parameter	Value
Experiment		
System repetition rate	$\nu _{\rm rep}$	80 MHz
Source efficiency	$r_c$	$1.11\times 10^{-3}$
Detector overall efficiency	$\eta _{\rm Det}$	37.5%
Dark count rate	$p_{\rm Dark}$	$1\times 10^{-7}$
QBER @ 0 km	$e_{0}$	0.9%
Channel loss (deployed)	$l_{\rm dep}$	4.0 dB
Channel loss (spool)	$l_{\rm sp}$	11.2 dB
Alice device loss	$l_A$	6.2 dB
Bob device loss	$l_B$	1.7 dB
Simulation		
Security parameter	$\epsilon _{\rm sec}$	$10^{-12}$
Correction parameter	$\epsilon _{\rm cor}$	$10^{-12}$
Error correction factor	*f*	1.16

## DISCUSSION

A polarization-encoded BB84 experiment was performed with single photons generated by the GaN defect-based SPS, which emits telecom photons at room temperature. With a base-choosing strategy to minimize the impact of PMD, a field trial over a 3.5-km deployed fiber with high PMD was demonstrated. We achieved a secure key rate of 585.9 bps with high stability for 7 h. The feasibility of a longer distribution was confirmed by another experiment over a 32.5-km spool. The secure key rate in both the experiments approached the GLLP limitation with current devices.

The main source of the QBER is the depolarization effect caused by PMD due to a relatively broadband spectral emission of the SPS. The fiber spool exhibited substantially lower PMD than the deployed fiber, suggesting that the SPS could also support polarization encoding over a longer fiber distance with reduced PMD. This could be achieved by either narrowing the linewidth of the SPS emission [48], switching to fiber models with lower PMD [41] or seeking proper PMD-compensating methods [49].

Compared to prior QKD experiments with room-temperature SPSs emitting at shorter wavelengths, we were able to achieve a higher secure key rate (under the same channel loss) that was approximately two orders of magnitude higher than in [34], but around two orders of magnitude lower than in [24] and [50]. Our contribution lies in the fact that we could expand the channel environment beyond the lossless free-space [34,50] and 27-dB free-space attenuator [24] to include a deployed fiber link with high PMD as well as a much longer fiber spool. Another way to mitigate the detrimental effect of PMD could be utilizing a time-bin encoding scheme, which we report in [36], although the setup incurred higher losses at the encoder and decoder, and required additional active temperature compensation. Other QKD experiments employing cryogenic SPSs [27,51] achieve key rates that exceed ours by two to three orders of magnitude, mainly due to higher brightness. As demonstrated in these works, the performance of our scheme in the future could also be enhanced by integrating photonic structures, which could significantly increase the brightness and improve the $g^{(2)}(0)$ and emission linewidth.

In conclusion, the results reveal the potential for room-temperature telecom SPSs to be applied in polarization-encoded QKD, and therefore pave the way for more practical implementations of SPSs on QKD neatly and easily.

## MATERIALS AND METHODS

### Polarization-encoded BB84 setup

For state preparation, a Sagnac-like polarization modulator [52] capable of a high repetition rate was employed, as illustrated in Fig. 1. As this is a proof-of-concept experiment, the modulator settings were chosen from a prepared file and not a quantum random number generator. The experiment was synchronized to a clock with an 80-MHz repetition rate.

The incoming photon wavepacket was separated into $|H\rangle$ and $|V\rangle$ arms by a polarization beam splitter (PBS) with equal probability. These arms were made from polarization-maintaining fiber and arranged such that the photons propagate simultaneously along the fast axis. Since the phase modulator is placed asymmetrically in the loop, the pulsed modulation signal only modified the clockwise or the anti-clockwise signal phase, generating a phase difference between $|H\rangle$ and $|V\rangle$. After being combined by the PBS, a series of polarization states $|H\rangle +e^{i\phi _V}|V\rangle$ could be created by controlling the electro-optical phase modification $\phi _V$. With $\phi _V$ calibrated to $0, \pi /2, \pi , 3\pi /2$, the polarization states $|D\rangle , |L\rangle , |A\rangle$ and $|R\rangle$ required by the BB84 protocol were generated.

The receiver was a passive basis-selection polarization measurement setup. A non-polarizing beam splitter was applied to randomly select a basis for measurement. The single-photon signals were measured with a superconducting nanowire single-photon detector (SNSPD), and their time of arrival was recorded by a time tagger [53].

### Key analysis

The length of the secure key could be given by [45]


(3) \begin{eqnarray*}
l=n_zA_z[1-h(Q_x+\delta )]-{\rm leak}_{\rm EC}-\log \frac{2}{\epsilon _{\rm sec}^2\epsilon _{\rm cor}}.
\end{eqnarray*}

This formula separates the sifted key into bit basis *Z* with length $n_z$ and phase basis *X* with length $n_x$ for security analysis, where the *Z* basis is used to generate the secure key and the *X* basis is employed to bound Eve’s information. In this work, we choose the DA basis as *Z* and the LR basis as *X*. Alice and Bob choose the bases with probabilities $p_x$ and $p_z$. Here *l* is the length of the secure key, and $A_z=1-{p_m}/{p_{\rm det}p_z}$ and $A_x=1-{p_m}/{p_{\rm det}p_x}$ correspond to the security leakage from the multi-photon emissions in the *Z* and *X* bases, respectively, where $p_m$ is the probability of multi-photon events Alice sends into the quantum channel and is upper bounded by $p_m\le {g^{(2)}(0)\mu ^2}/{2}$, while $p_{\rm det}$ is the detection probability. The mean photon number *μ* is estimated with the long-term average counts of the SPS brightness, repetition rate and SNSPD efficiency calibrated with a 1310-nm weak coherent laser. We denote by $h(q)$ the binary Shannon entropy function, $Q_x={e_x}/{A_x}$ is the single-photon QBER of the *X* basis, assuming that all multi-photon states are untrustable,


\begin{eqnarray*}
\delta =\sqrt{\frac{(n_z+n_x)(n_x+1)}{n_zn_x^2}\ln \frac{2}{\epsilon _{\rm sec}}}
\end{eqnarray*}

is the statistical fluctuation of QBER estimation, $\epsilon _{\rm cor}$ is the security parameter in error correction, and $e_z$ and $e_x$ are the experimentally observed QBERs in the *Z* and *X* bases. Finally, ${\rm leak}_{\rm EC}=fh(e_z)n_z$ represents the information leakage in error correction.
